# Testing ethical impact assessment for nano risk governance

**DOI:** 10.12688/openreseurope.16194.1

**Published:** 2023-10-10

**Authors:** Ineke MALSCH, Panagiotis Isigonis, Evert Bouman, Antreas Afantitis, Georgia Melagraki, Maria Dusinska

**Affiliations:** 1 Malsch TechnoValuation, Utrecht, 3582EX, The Netherlands; 2 Ca' Foscari University of Venice, Venice, Veneto, Italy; 3 NILU, Kjeller, Norway; 4 Nova Mechanics, Nicosia, Cyprus

**Keywords:** nanomaterials, ethics, risk governance, ethical impact assessment

## Abstract

Risk governance of nanomaterials and nanotechnologies is traditionally mainly limited to risk assessment, risk management and life cycle assessment. Recent approaches have experimented with widening the scope and including economic, social, and ethical aspects. This paper reports on tests and stakeholder feedback on the use of ethical impact assessment guidelines and tools adapting CEN Workshop Agreement part 2 CWA 17145-2:2017 (E)) to support risk governance of nanomaterials, in the RiskGONE project.

## Introduction

Increasingly, life cycle, or value chain based, models are used to explore potential environmental, economic, and social impacts of emerging technologies during research and innovation projects. One example is the application of (social) Life Cycle Assessments (LCA) for quantification of environmental or labour footprints associated with a product, process, or service. While some research and innovation projects raise ethical issues, ethical impacts are not generally considered in life cycle models. This paper reports on tests of guidelines and tools supporting stakeholders engaged in dialogue on risk governance of nanomaterials to identify and assess ethical impacts alongside more traditional risk assessment indicators and impacts related to environment, economy, and society ( Riskgone D3.6 (2020)). While intended to be used by stakeholders engaged in risk governance of nanomaterials, the guidelines and tools may also be useful for other stakeholders such as companies or project coordinators leading research or developing nanotechnology-enabled products ( Malsch *et al.*, 2021b). The developed guidelines and tools for ethical risks and benefits assessment for nanomaterials are based on the Ethical Impact Assessment (EIA) procedure as defined in the CEN Workshop Agreement part 2 CWA 17145-2:2017 (E) on Ethical Impact Assessment (EIA) ( CEN, 2017). Building upon an earlier concept paper ( Malsch *et al.*, 2020), the present paper reports on tests and stakeholder feedback on the use of these ethical impact assessment guidelines and tools to support risk governance of nanomaterials. The tools were tested on several case studies and discussed with stakeholders. The aim is to give a step-by-step explanation of the ethical impact assessment procedure, to demonstrate the practical use of the online ethical impact assessment tools ^1^, and to discuss possible improvements to increase the relevance of the tools for stakeholders in risk governance of nanomaterials.

## Methods

**Figure 1.**
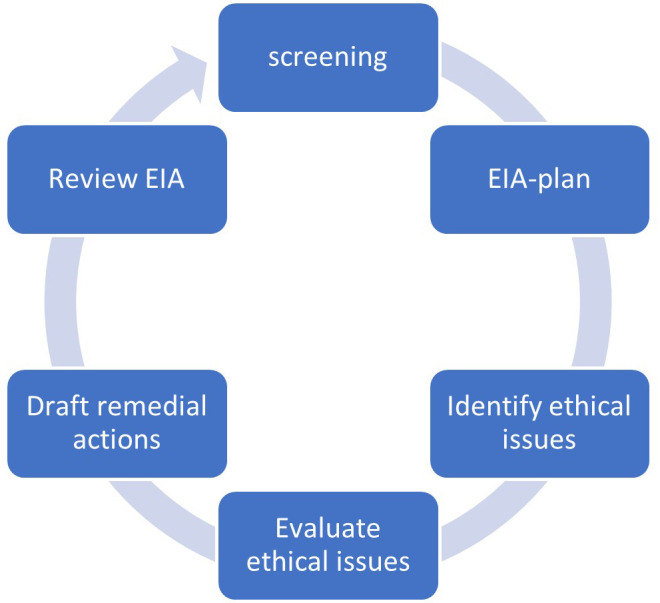
The EIA procedure as prescribed in the CEN pre-standard on ethical impact assessment ( CEN, 2017)

Online tools support users in performing the six-step EIA procedure (screening, drafting an EIA-plan, identifying, and evaluating ethical issues, drafting remedial actions, and review of the EIA) depicted in Figure 1. These tools and guidelines developed in the project facilitate ethical impact assessments of case studies. The outcomes of these tests are used for generating input into further development of the tools. Six case studies on nanomaterials were used for testing the tools and guidelines. One case study focused on the case of the risks and benefits of utilising nanomaterials in wastewater remediation in developing countries. The results of this case study are reported in the book chapter Malsch *et al.* (pending publication). Another case study focused on the ethical risks and benefits associated with utilising nanomaterials for solar energy in developing countries. A third explored ethical impacts of nanomaterials in dentistry, a fourth addressed impacts of nanomaterials in tyres, a fifth targeted issues raised by using ZnO nanoparticles for combatting citrus greening, and the sixth compared ethical issues of sharing or not sharing nanosafety data.

Most case studies were chosen as they reflect the use of nanomaterials in different application areas, rather than a single nanomaterial which can have many uses. Two applications ( *i.e.,* wastewater remediation and solar energy) aim to solve current problems related to environmental pollution associated with human activity serving human needs. Respectively, the needs for clean and fresh water, and the need for a clean and green energy supply. The benefit and, potentially, urgency of the intervention is reinforced by situating the cases in developing countries. Issues of using nanomaterials in a healthcare context were at the core of the case study of nanomaterials in dentistry, and the case study on ZnO nanoparticles highlighted food and agricultural ethical issues. The case study on nanomaterials in tyres built upon earlier discussions at the OECD ( OECD, 2014). The case study on sharing nanosafety data addressed discussions about responsible nanosafety research.

In the following sections, the 6-step EIA procedure and the case study outcomes within each step are described, and results of discussions with stakeholders presented.

## Step 1: screening potential ethical impacts

The self-assessment of ethical issues prescribed in the CEN pre-standard is limited to a checklist of nine categories of negative ethical impacts: Health, Privacy, Liberties, Equality, Common good, Environment, Sustainability, Military dual use, and Misuse. These categories span a wide range of ethical issues, making the tool suitable for screening many kinds of nanomaterials for any conceivable application. For example, nano-enabled wastewater cleaning in developing countries raises different issues from nanomaterials in dentistry, but the tool is applicable to both.

In the online tool developed by us, the prescribed procedure was programmed to determine whether a full-scale ethical impact assessment is needed, and to determine the scope of such an EIA into an online threshold analysis decision tree ^2^. The user selects relevant ethical impacts and indicates the severity of each point (1 = minor; 2 = moderate; 3 =medium; 4= high; 5=severe). The output is one of four different recommendations: no EIA, or a small, medium, or large-scale EIA. No EIA is needed if none of the identified issues is more than minor (1). In cases where the self-assessment concludes that no EIA is needed, the user should consult an external ethicist to confirm this. A small EIA is needed if less than three issues are identified and at least one is moderate (2), or at least three issues are identified, but none more than moderate (2). A medium EIA is needed if three or four issues are identified and at least one is high or severe (4–5), or at least five issues are included and at least one is moderate or medium (2–3). A large EIA is needed if at least five issues are identified and at least one is high or severe (4–5).

This ethical impact assessment will be integrated in a multicriteria risk-benefit assessment as part of a larger risk benefit assessment procedure covering health and safety, economic, social, and environmental aspects. To support the self-assessment of ethical risks as well as benefits, another risk-benefit decision tree was developed with the same checklist, where the user can select and estimate the size of ethical risks as well as benefits. The output of the ethical impact assessment screening of nanomaterials for wastewater remediation, ZnO nanoparticles combatting citrus greening, nanomaterials in dentistry, and sharing nanosafety data were small-scale EIAs, and for solar photovoltaics and nanomaterials in tyres that no EIA was needed. However, to complement studies by partners on other risk assessment studies of nanomaterials in tyres, a complete small-scale EIA was nevertheless performed on this case study.

**Figure 2.**
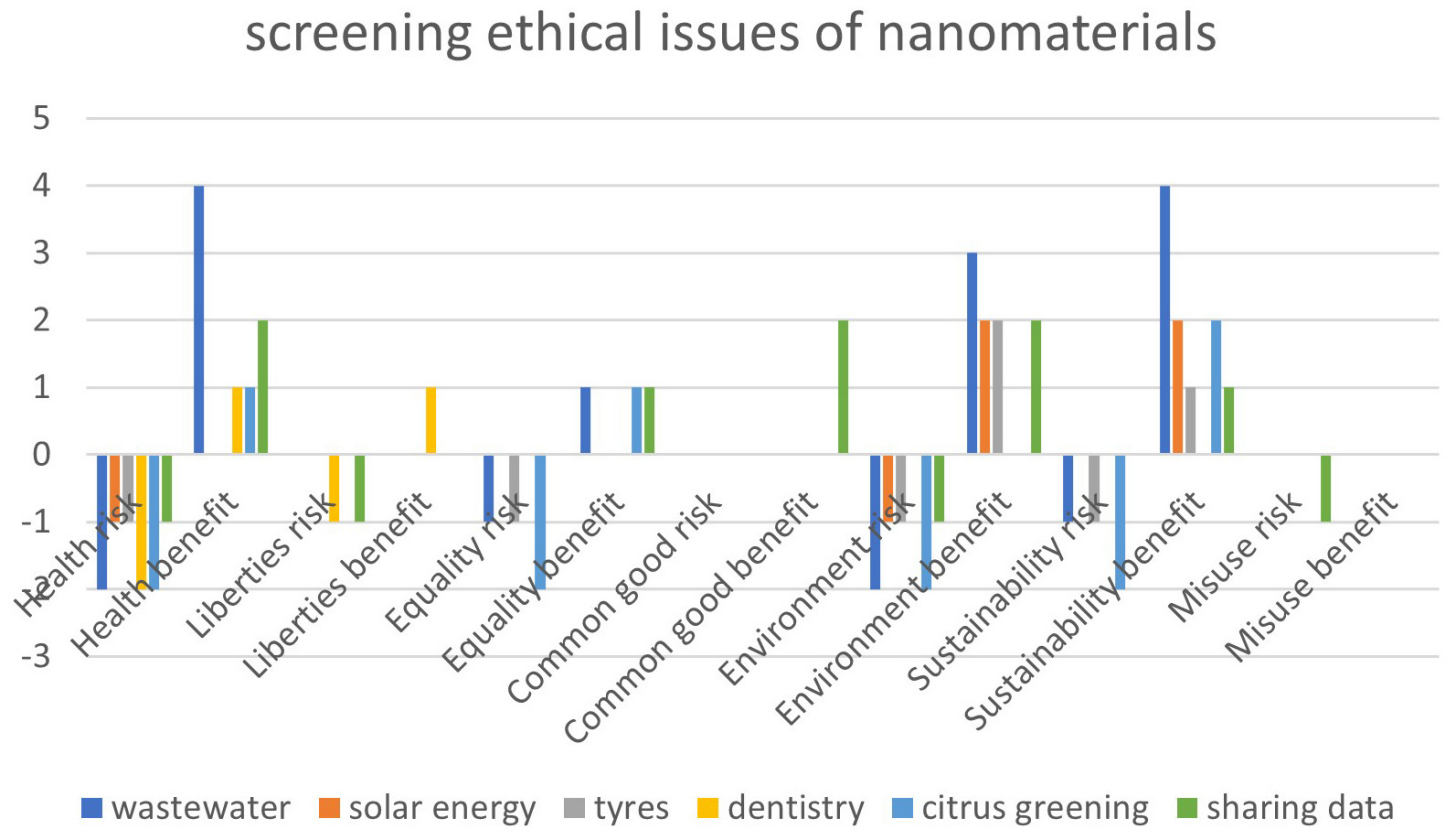
Output of self-assessment screening of ethical risks and benefits of nanomaterials for wastewater remediation in developing countries, for nanomaterials in solar photovoltaics, for using nanomaterials in tyres and dentistry, ZnO nanoparticles for combatting citrus greening, and sharing nanosafety data.

Comparing the results of the self-assessment shows that the tool helps focusing further analysis on relevant ethical issues. Not all categories were relevant to the selected cases. In none of them, ethical impacts on privacy or military dual use were expected, which is understandable given the focus of the overall risk governance framework on civil applications of nanomaterials raising environmental, health and safety concerns. In all cases, minor or moderate health related ethical risks were foreseen, related to nanosafety issues, but in the case of dentistry also related to biomedical ethical issues. In four cases, these risks were balanced by foreseen benefits to health, for wastewater remediation these were deemed strong, for sharing nanosafety data moderate, and for dentistry and citrus greening minor. Minor to moderate environmental risks were expected in all cases except dentistry, which were balanced by moderate to strong environmental benefits in four of these cases (wastewater, solar energy, sharing data, and tyres). Three cases raised minor or moderate equality related concerns (wastewater, tyres, and citrus greening), which were balanced by minor expected benefits for wastewater, sharing data, and citrus greening. While three cases raised minor or moderate sustainability issues (wastewater, tyres, and citrus greening), sustainability was deemed to benefit strongly by applications of nanomaterials in wastewater remediation and solar energy, moderate by applications combating citrus greening and minor by applying them in tyres and by sharing data. In dentistry and sharing data, minor risks for liberties were foreseen, balanced by minor benefits in dentistry. Sharing data raised minor concerns about potential misuse but offered moderate positive prospects for contributing to the common good. The output of the risk-benefit tools for the cases is depicted in Figure 2.

### Stakeholder testing of screening tools

The output of the screening of ethical impacts in the case-study on wastewater was discussed during an online meeting of predominantly nanosafety experts. Because the session was limited to 30 minutes, the participants were asked to give their quantified estimate of the severity of ethical issues identified in literature on a five-point scale of 1=minor to 5=severe. They were also asked to give such estimates of the strength of ethical risks and benefit at stake in the case study. The severities indicated by the participants (3–4) were a lot higher than of the ethics expert doing the original impact assessment (1–2). Due to time constraints, it was not possible to find out if this was due to different calibration of the scales or to substantial differences in evaluations of the ethical risks and benefits (see also Malsch *et al.*, 2021b). The raw data on the responses of these stakeholders is included in Malsch (2023).

During a later demonstration of the EIA tools on the case study of nanomaterials in dentistry, organised by NanoCOMMONS on 18 November 2021, three groups of participants tested the self-assessment screening tool ^3^. All three groups agreed on the need for a medium scale EIA, while the ethics expert had considered a small-scale EIA to be sufficient. In addition, the issues identified by the three groups differed, as indicated in Table 1. The raw data on the responses of these stakeholders is also included in Malsch (2023). 

**Table 1.** Responses of three groups during stakeholder meeting at NanoCOMMONS training on 18-11-2021. Scale: 0=no, 1=minor, 2=moderate, 3=medium, 4=high, 5=severe.

Category	Group 1	Group 2	Group 3
Health	3	1	3
Privacy	0	1	0
Liberties	0	0	1
Equality & social justice	3	0	2
Common good & well being	3	0	2
Environment (waste)	3	2	2
Sustainability	0	3	3
Military dual use	0	0	0
Misuse	0	0	0

During the subsequent discussion, participants remarked that risk perceptions are personal, and never give similar results. Qualitative assessment of outputs by different stakeholder groups including regulators and NGO-representatives, or patients, could generate interesting results. Before discussing options for improving the EIA tools, the additional tools guiding the user through a full-scale EIA are presented (step2–6).

## Step 2: drafting EIA plan

If performing a full-scale EIA is deemed necessary, the user should prepare an EIA plan, which is proportionate to the size of the EIA. This includes selection of sources and methods for identifying and evaluating ethical impacts, drafting remediation measures, and review of the EIA by an independent ethicist. The online EIA tool has different online formats for planning a small, medium, or large-scale EIA, which the user can fill in and download as a pdf. ^4^

To test the EIA plan tool, simplified EIA plans were developed for case studies of nanomaterials in wastewater remediation, ZnO nanoparticles for combatting citrus greening, nanomaterials in tyres and in dentistry, and for sharing nanosafety data. These plans were limited to desk research by one ethicist of open access literature and online sources, combined with discussions with partners in similar research projects on risk governance of nanomaterials and participants in events where results were presented. In real life cases, larger EIA teams may need to be formed and additional resources may need to be budgeted for access to literature and for organising foresight activities and consultations with broader groups of stakeholders and experts. An external ethicist should review the plan before starting the EIA process.

The online EIA tools for identifying and evaluating ethical issues and for drafting remedial actions only support performing a small-scale EIA. Medium or large scale EIAs require the collaboration of larger teams, more resources, and more different methods for identifying ethical impacts. Since most applications of nanomaterials are unlikely to raise severe ethical issues and hence require more than a small-scale EIA, supporting larger EIA studies is out of the scope of this project. Larger-scale EIAs have been performed in other projects such as SIENNA. ^5^

## Step 3: identifying ethical impacts

The first substantial activity in the EIA process is the identification of relevant ethical impacts to the case under study. This step may be performed by junior staff without ethics training, but at least some understanding of relevant ethical issues is recommended. This part of the research is supported by several online tables on the webpage *Identification of Ethical Impacts*. ^6^ The work starts with collecting and reviewing ethics literature by the ethicist or EIA team. In the wastewater, tyres, dentistry, and sharing data case studies, relevant literature was identified through searching a shortlist of journals and internet repositories of grey literature where ethical aspects of these applications were discussed. The corresponding literature list was included in the *References* table. The EIA team analysed the collected literature using the online checklist on the same webpage. Table 2 summarises the more detailed issues identified in literature for four cases.

**Table 2.** Identifying ethical issues in literature. Regarding nanosafety data, ethical impacts of sharing and of not sharing it were identified.

		Wastewater	Tyres	Dentistry	Data
Health-related	Public health & safety	x	x	x	X
	***Applied in healthcare context***			x	
***Liberties***	***Autonomy***			X	
	***Human bodily integrity***			X	
	***Intellectual property***				X
***Equality***	***Distribution of risks & hazards***	x	X		x
	***People in developing countries***	X			X
	***Future generations***		X		
	***Unbalanced economic resources***				X
***Common good***	***Well-being of groups in society***				X
Environment	Water, land, harmful waste	x	X	x	X
***Sustainability***	***SDG12 (sustainable consumption & production)***		X		X
	***SDG13 (climate change)***		x		
***Misuse***					X

To avoid overlap with traditional nanosafety studies analysing environmental, health and safety risks, the analysis of ethical issues should henceforth be focused on complementary issues, highlighted in the table. To end this step, the results of the desk research were summarised in the online *report on the identification of ethical issues* and downloaded as a pdf for further analysis.

While the CEN pre-standard also calls for performing horizon scanning or another foresight study, this was not deemed necessary because sufficient issues were addressed in the identified literature. In addition, the CEN pre-standard requires stakeholder engagement as part of the identification of ethical issues and again during evaluation and drafting remedial actions. To limit the burden on the time of such stakeholders, all the activities of the EIA team on identification and evaluation of ethical issues and in drafting recommendations was combined in one package and the results of all three steps were discussed together in one online stakeholder consultation for each case.

## Step 4: evaluating ethical impacts

The evaluation of the identified ethical impacts is supported by several online tables on the webpage *evaluation of ethical impacts*. ^7^ While the identification of ethical issues in step 3 focused on negative ethical impacts, the evaluation step broadens out again to analyse negative as well as positive ethical impacts. The reason is that the evaluation includes balancing impacts on different identified ethical principles, where the consideration of potential ethical benefits as well as risks is needed. This evaluation assumes at least some training in philosophical ethical theories and in applied ethics. The ethicist should start by updating the literature list with references to relevant ethical principles and theories. For example, in the case where nanotechnology is used in wastewater remediation in developing countries, the precautionary principle ( COMEST, 2005) water ethics framework ( Liu *et al.*, 2011) and global code of conduct for research in resource-poor settings ( Global code, 2018) are applicable. In the case of nanomaterials in dentistry, the Dental Ethics Handbook offered relevant guidance ( American college of dentists, 2016).

The ethicist should clarify the relevant ethical principles and values by applying ethical theories to the identified issues and by analysing what light the key ethical concepts in those theories shed on the case under study. The first online form on this webpage supports this analysis. In the wastewater case, for example, during the development and testing of the technology, precaution, the selection of affordable local resources and participation are more relevant, as well as the principles of the global code of conduct for research in resource-poor settings (fairness, respect, care, and honesty). In later stages, where the systems will be used on a large scale and end up in waste processing, the human right to water and other water ethics principles are dominant, while the use of affordable local resources and participation remain relevant.

The ethicist should then assess the degree to which each identified ethical value or principle is likely to be violated or benefited in the expected ethical impact. This analysis is supported by an online decision tree in the second table on the webpage (illustrated for wastewater in Table 3).

**Table 3.** Example of assessing the likelihood and intensity of violation of ethical values of using nanomaterials in wastewater remediation in developing countries. (1 = minor; 2 = moderate; 3 =medium; 4= high; 5=severe). Adapted from: Malsch *et al.*, pending publication.

Ethical risk	Identified principle or value	Ethical benefit
1	Water ethics: equity	4
1	Water ethics: multiple and beneficial use of water	4
1	Water ethics: users and polluters pay principle	4
2	Fairness, respect, care, honesty (global code)	1
2	Precaution	2

The third table on the webpage supports the analysis of trade-offs between different relevant ethical principles or values. During the development of the technology in the wastewater case in some regions without access to clean water, trade-offs may occur in the short to medium term between the precautionary principle, which could lead to delays in development of innovative solutions, and the human right to clean water (see Table 4). Other trade-offs in this case are more related to conflicting interests of different water users, including industry, agriculture, and consumers. Those trade-offs must be tabled in participatory dialogues engaging all stakeholders.

**Table 4.** Example of remediating value conflicts in the wastewater-case.

Value 1	Value 2	Description of the value conflict	Proposed remediation
Human right to water	Precautionary principle	In some target countries, many consumers currently do not have access to clean water. The nanotechnology- enabled wastewater remediation system is therefore urgently needed. However, the precautionary principle calls for taking risk management measures to address uncertain risks of nanomaterials which may be released in the cleaned water, which could delay implementation of the solution.	While the human right to water is more fundamental than the precautionary principle, the project partners do not have the primary responsibility for it. The consortium could engage with the national government or donors to ensure access to clean drinking water from other sources during the project and invest sufficient resources in investigating potential release and health or environmental impacts of the nanomaterials used in the wastewater remediation system.

In cases where conflicts between fundamental values are at stake, the ethicist should find ways to solve such value conflicts by following up to five rules of thumb prescribed in the EIA guidelines. In the dentistry-case, a value conflict was identified between the freedom and intellectual property rights of producers and the biomedical ethics principles and liberties of the patients and dentists. The first rule of thumb is: *‘If one conflicting value is fundamental and the other is not, prefer the fundamental value. If both are fundamental, keep the value conflict and apply rule of thumb 2.’* In this case, both conflicting values are fundamental, requiring application of the second rule of thumb: *‘Assess the degree of violation of conflicting fundamental values and choose the action that least compromises a fundamental value.’* To resolve this, a precautionary approach was proposed to complement a regulatory gap in nanoregulation (at least at the time of publication of the analysed literature), exploring pros and cons of different approaches used in cases documented in literature.

To conclude the evaluation of identified ethical issues, all information included in the tables on the corresponding webpage should be downloaded again as a pdf for further analysis and for stakeholder and expert consultations.

## Step 5: remediating ethical issues

The full-scale EIA should result in practical recommendations for remediation of the identified ethical issues. The formulation of these recommendations is supported by online tables on the webpage *Remedial actions for EIA ^8^*. The first step is to collect recommendations from similar projects. In the wastewater case, for example, two projects were identified recommending comprehensive ethical frameworks for water management and research in resource poor settings, which are well-balanced frameworks for addressing societal and organisational impacts. The societal recommendation is to implement the UNESCO water ethics framework ( Liu *et al.*, 2011) in national regulations or policies. The organisational recommendation is to apply the code of conduct for research in resource-poor settings ( global code, 2018).

After this, the EIA team should formulate its own societal and organisational recommendations for the case under study. Two online tables guide them through the process, which relies strongly on discussions of draft recommendations with stakeholders. The drafted recommendations and report of stakeholder engagement should then be included in a report and downloaded as a pdf. In the wastewater case, discussion of the recommendations was combined with discussions on the identified issues and result of the ethical evaluation. Due to time constraints, little stakeholder feedback was received.

## Step 6: external review of Ethical Impact Assessment

An essential element of a credible Ethical Impact Assessment is the mandatory check of the results and of the followed procedure by an external ethicist. The webpage *Review of EIA ^9^* includes guidance for such external review. The peer review of this paper is considered to take the place of such an external review. In real life ethical impact assessments, sufficient resources and the engagement of a qualified external ethicist must be foreseen during the planning of the EIA.

## Discussion of improvements of the EIA tools

After testing the online tools on case studies and discussing the demonstrated tools with partners in several projects on nanosafety (reported in Malsch *et al.*, 2021a; Malsch *et al.*, 2021b), the following conclusions can be drawn on the expected users of the tools, ethically sensitive applications of nanomaterials and relevant ethical issues related to nanomaterials.

### Scope of the analysis

Nanosafety experts commented on the choice of nanomaterials impacts in the case-study on wastewater. The case study was based on a review of different kinds of nanomaterials used in wastewater remediation including carbon and graphene-based materials, metal organic frameworks and metal oxides and composites ( Kokkinos *et al.*, 2020). According to participants, the case study should be focused on TiO2 because there is mounting evidence of nanosafety issues of those materials, and not of other nanomaterials used in wastewater remediation. Nanosafety experts discussing the dentistry case recommended that the EIA tools should be used incrementally, starting with simple cases, and advancing to more complex cases. From simple to complex materials, or from production of a material to a complex use case of composites incorporating nanomaterials such as dentistry.

### In which context can the EIA tools be used?

The Ethical Impact Assessment pre-standard ( CEN, 2017) underlying the EIA tools was developed by European partners in the SATORI project ^10^. Discussions with African participants in two online events of the analysis of ethical issues of nanomaterials in wastewater remediation in developing countries suggest that the tools in their present form may not be immediately suitable for non-European contexts. In the ANSOLE DAYS & BALEWARE 2021 online conference, the participants were mainly academic experts in sustainable energy in Africa and other countries, raising the question whether performing the ethical impact assessment of nano-enabled wastewater remediation was relevant in the African context. During the International Water-Climate Summer School at North-West University, Mahikeng Campus, North-West Province, South Africa, on 4 – 18 October 2022, social scientists discussed ethical dilemmas in their studies of local communities involved in water and climate research. The focus was mainly on research integrity issues, including interpretation of the rules protecting the rights of human participants, avoiding bureaucracy, and on how to safeguard scientific quality, while taking responsibility for the impact of policies inspired by the research results on the livelihood of the participants.

### Who should use the EIA tools?

Nanosafety experts most often expected ethicists to use the EIA tools (nine of the thirteen respondents in a small survey reported in Malsch *et al.*, 2021a). See also Malsch, 2023. This is in accordance with the original intentions of the partners in the SATORI ^11^ project who developed the underlying CEN pre-standard for use by an Ethics Committee ( CEN, 2017). Testing the online EIA tools on several case studies confirmed that the participation of at least one (junior) ethicist in the team performing a full-scale ethical impact assessment is needed to ensure sufficient quality of the assessment. However, eight of these nanosafety experts expected industry and seven government to use the tools. Testing the EIA-tools also indicates that industrial companies and government agencies could use the tools for assessing potential ethical issues of nanoproducts as part of a broader risk governance framework. They could employ interdisciplinary EIA-teams including at least one ethicist.

### Potential applications of the EIA tools

Seven of fourteen responding nanosafety experts expected their organisation or themselves to use the EIA tools as an integral part of risk governance of nanotechnology, for which the tools were developed. Four respondents would use the EIA tools for screening ethical impacts of a product or project, suggesting that the self-assessment screening tool (step 1) could be appropriate for non-expert use. After testing the tools on several cases, this potential use is confirmed, and more concrete instructions were developed for non-expert users wishing to perform a self-assessment of potential ethical issues of nanomaterials under development for specific applications. No respondents expected to use the full set of EIA-tools to guide a full ethical impact assessment, confirming that users should at least employ one ethicist for this. The screening tool is deemed useful enough for non-experts to perform an initial self-assessment of potential ethical issues. Finally, three respondents do not expect to use the EIA tools.

Nanosafety experts participating in the NanoCommons training on 18 November 2021 suggested another application of the self-assessment screening tool: to ask different stakeholder representatives to estimate their perceived severity of ethical issues of a given nanomaterial or use case and then discuss the differences between the groups. Developing the tool further for such a purpose could be the topic of a follow-up project.

### Which ethical issues should be addressed?

Testing the EIA tools on several applications of nanomaterials indicates that not all categories in the screening tool are relevant to risk governance of nanomaterials, and not all ethical concerns perceived by stakeholders may fit comfortably in one of these categories. To improve the fit of the checklist to user needs, nanosafety experts were asked which nanotechnologies or nano-enabled products raise ethical issues, receiving thirteen responses. While nine or ten answers suggested nanotechnologies or nano-enabled products, others addressed the fate during the life cycle or impacts of nanotechnology ( *e.g.,* on humans or the environment). ‘Climate change’ could refer to applications of nanotechnology to remediate climate change or effects of the use of nano-enabled products on climate change. The former was assumed. Several answers combined two categories ( *e.g.,* food or health combined with sensors). Food-related applications are currently not explicitly addressed by the EIA-screening checklist, while the other applications are more clearly targeted by some of the pre-existing categories (health, privacy, and sustainability). The responses were very brief key words, and it was not always clear what was meant.

The ethical categories which are currently included in the EIA-screening tool do not quite match the suggested ethical issues respondents consider relevant to risk governance of nanotechnology. While privacy, equality, environment, and the sustainable development goals are both included in the checklist and mentioned by respondents, the latter did not mention specific healthcare related issues, liberties, the common good, dual use, and misuse aspects. Related to health, they suggested health impacts of nanotechnology, and related to sustainability, materials sourcing was mentioned. Some of the mentioned issues are already addressed in other modules of the risk governance framework for nanotechnology, especially impacts on human health and the environment. Furthermore, respondents mentioned other issues and values which are commonly addressed in discussions related to nanosafety, such as traceability, transparency, accessibility of confidential business information, and trust. Finally, ethical (researcher and corporate social responsibility) codes were mentioned. These codes combine several ethical principles and values and are relevant as reference documents. In the analysis of the wastewater case, such reference documents were used as sources for relevant principles and values ( Global code, 2018; Liu *et al.*, 2011).

**Figure 3.**
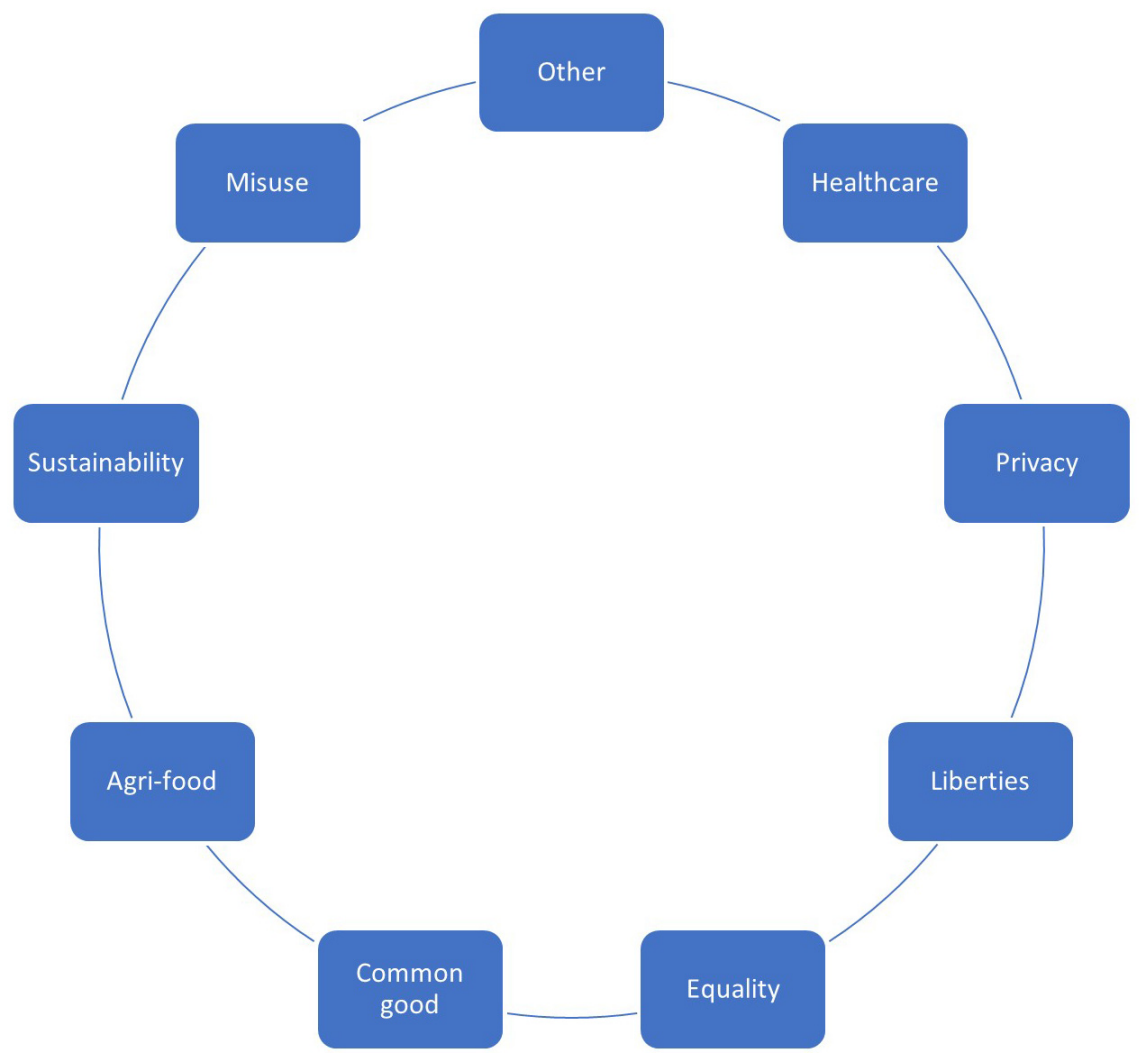
Suggested revised EIA screening checklist.

Follow-up discussions with stakeholders and tests of the EIA tools on case studies suggest that overlap of the EIA with traditional risk assessment and life cycle analysis of nanomaterials should be avoided, because this will be done by experts in other fields. While in the screening stage, users may still tick environmental, health and safety issues, a subsequent full-scale EIA should be focused on complementary ethical issues, such as healthcare ethics, privacy, liberties, equality, common good, agri-food ethics, sustainability, and misuse. Categories for a possible revised checklist for EIA in the nano risk governance framework are depicted in Figure 3. To fit in the existing decision tree format, the checklist has again nine categories. Other issues could be environmental, health and safety issues, military dual use, artificial intelligence, or another issue relevant for the screened material and product in its societal context. The user should briefly describe each issue and estimate the severity (1–5).

### Improving user-friendliness

Stakeholders testing the screening tool recommended to explain the interpretation of each category more extensively for non-expert users. Four respondents commented on the question how the Ethical Impact Assessment tools can be improved. One was satisfied with the tools, suggesting that users should “review the categories and criteria to establish the category that one thinks corresponds to it.” Two respondents wanted more specific and direct information links to relevant guiding documents and articles. One wanted simpler questions, recommending “…to be always as specific as possible in the questions,” and formulating more subjective answers, such as: “I am severely concerned, I do not consider this relevant,....” Another proposed giving “maybe more examples in case of open questions, so that the user gets hints how the questions have to be answered (could be answered).”

These respondents also made suggestions on information to be offered to guide a non-expert user through using the tools, including Information on benefits and unwanted effects at layman's level links with more information and examples, and a glossary or simply reformulated questions.

One respondent suggested removing the category ‘military dual use’, explaining: “One of my functions is risk management and therefore there are aspects that I have clear, but it seems to me that asking if nanomaterials can be used in military matters is not the way to be approached because if they only serve to improve logistics or information, they have no impact on the civilian population nor will they cause harm to soldiers.”

Another respondent proposed “eventually test[ing the tools] on different stakeholder groups: users, regulators, NGOs.” This was in line with the earlier mentioned suggestion to use the screening tool to identify differences in value perceptions as a basis for stakeholder dialogue.

## Conclusions

The online EIA tools developed in RiskGONE offer practical guidance and support for professionals and ethicists who want to explore potential ethical issues of the use of nanomaterials and nanotechnologies in products for a wide variety of applications in different societal contexts. The methodology we explained in this paper has been tested on several case studies. For the applications of nanomaterials in solar energy and in tyres, no significant ethical impacts were identified in the preliminary screening stage, over and above the traditional environmental, health and safety and life cycle issues. Therefore, no full ethical impact assessment was deemed necessary beyond risk assessment and life cycle analysis. For sharing nanosafety data, the application of nanomaterials in photocatalytic decontamination of wastewater, nanomaterials in dentistry, and ZnO nanoparticles to combat citrus greening, moderate ethical issues were identified, calling for a small-scale ethical impact assessment by one (junior) ethicist in consultation with stakeholders. The in-depth results of some of these case studies will be reported in separate papers.

The discussions with different groups of stakeholders resulted in concrete proposals for online EIA screening tools which are more focused on specific ethical issues raised by the nanomaterials and applications targeted by risk governance, for improving the user-friendliness of the online EIA tools, and for using the tools to support stakeholder dialogue on value conflicts at stake in risk governance of nanomaterials.

## Ethical approval

The work was aimed at testing and improving the methodology for ethical impact assessment ( CEN CWA 17145-2:2017 (E)) through desk research and did not involve collecting, storing, or processing any personal data. Some anonymous responses from stakeholders participating in online events organised by others were used to test the methodology. All responses were collected anonymously from the start and no names, e-mail addresses or other personal data from the participants were handled for the work reported in this paper. The work was done in accordance with the data minimisation principle, and no citizens or vulnerable persons were involved. According to the Dutch Data Protection Authority, no prior ethical approval is needed in this case: https://autoriteitpersoonsgegevens.nl/.

The explanation in Dutch of the foundations for the GDPR is given here: https://autoriteitpersoonsgegevens.nl/themas/basis-avg/avg-algemeen/grondslagen-avg-uitgelegd.

## Data Availability

Anonymous raw data were collected through the Mentimeter polls in the online stakeholder workshops on 26–27 January 2021 and 14 April 2021 of the NMBP-13 projects (Gov4Nano, NanoRIGO and RiskGONE), and the NanoCommons training on 18 November 2021. These data are stored in Zenodo. Responses from participants in ANSOLE DAYS & BALEWARE 2021, 4–5 February 2021 http://ansole.com/ and in the International Water-Climate Summer School at North-West University, Mahikeng Campus, North-West Province, South Africa, on 4 – 18 October 2022 were not recorded. The presenter, Ineke Malsch, took notes of the remarks of participants. Zenodo: Dataset annexed to: "Testing ethical impact assessment for nano risk governance". https://doi.org/10.5281/zenodo.8095552 ( Malsch, 2023). This project contains the following underlying data: Joint NMBP13 Tools meeting26012021.pdf NMBP13_Joint_Conference_Poll_Results_Workshop_Ethical_Impact_Assessment_Tools.pdf NMBP13NRGF26012021ethics.xlsx Survey 18 Nov 2021.xlsx Data are available under the terms of the Creative Commons Attribution 4.0 International license (CC-BY 4.0).
