# Supplementary material for: Testing ethical impact assessment for nano risk governance
Source: Open Res Eur. 2023 Oct 10;3:170. [Version 1] doi: 10.12688/openreseurope.16194.1 (PMC11211730; doi:10.12688/openreseurope.16194.1)
Supplement: Peer review history

# Reviewer report: Reviewer response for version 1

**DOI:** 10.21956/openreseurope.17482.r35823

**Published:** 2023-11-21

**Authors:** Erich Griessler

**Affiliations:** 1 Institute for Advanced Studies, Vienna, Vienna, Austria

The article presents an ethical impact online tool to assess ethical risks and benefits of nano technology which might also be used in other emerging technologies.

 Although in general the framework and the process are well described and illustrated by practical examples of six case studies, some information is missing that would make the reader better understand the development and status of the framework. In general, the methods and process of how the tool has been developed, how the different steps were developed, how and by whom the online tool has been tested and discussed with external stakeholders, is not sufficiently described, e.g.:

- Page 4 mentions an online meeting of “predominantly nano safety experts”. It would be interesting to know how many they were, the selection criteria, why the mentioned time constraints existed and whether these might have an impact on the rating of the experts.
- Page 5 mentions “three groups of participants” at NanoCOMMONS. How big were these groups, how where they composed, what was the format of discussion?
- Page 6 mentions an “online consultation”. Who was consulted? How were the stakeholders selected? What was the process of the stakeholder consultation?
- Page 8 mentions “nano-safety experts”. Who were they? How many were they? How were they selected? What was the selection criteria? How was this process organized?
- Page 8 mentions “African experts”. Who were they? How many were they? How were they selected? What was the selection criteria? How was this process organized? The same question are applicable to “social scientists” mentioned on the same page in the text.
- Page 9 mentions follow up discussions with stakeholders and test. An explanation would be needed about how this was done.

The article conveys an important and extraordinary role to ethics experts (e.g., p. 6, p. 7, p8.) without explaining why. Handing over the ethics review primarily to an ethicist/external ethics reviewer might carry the danger that the ethics assessment gets an isolated activity that is not well connected to scientific and technical parts of the project. This also mirrors in one of the responses of the stakeholders that ethicists should do an ethics review. The authors might comment on the risk that ethics review might become an activity that is not well integrated in the overall project and how to better integrate ethics assessment in the development of emerging technologies.

Is the rationale for developing the new method (or application) clearly explained?

Partly

Is the description of the method technically sound?

Partly

Are the conclusions about the method and its performance adequately supported by the findings presented in the article?

Partly

If any results are presented, are all the source data underlying the results available to ensure full reproducibility?

Partly

Are sufficient details provided to allow replication of the method development and its use by others?

Partly

Reviewer Expertise:

Science Technology Studies, Responsible Research and Innovation

I confirm that I have read this submission and believe that I have an appropriate level of expertise to confirm that it is of an acceptable scientific standard, however I have significant reservations, as outlined above.

# Response

**Published:** 2024-01-10

**Authors:** Neelina Hermina MALSCH

Thank you for these comments. In response, we tried to improve the paper. The role of ethics experts and experts from other disciplines was addressed in the conclusions. We added more information about the numbers and backgrounds of the participants in the stakeholder engagement events, and how the events were performed.

# Reviewer report: Reviewer response for version 1

**DOI:** 10.21956/openreseurope.17482.r35817

**Published:** 2023-11-21

**Authors:** Araceli Sánchez Jiménez

**Affiliations:** 1 Instituto Nacional de Seguridad y Salud en el Trabajo, Barakaldo, Spain

The paper deals with a very relevant issue that is the ethical use of nanomaterials in different applications.

 The aim seems to develop a tool for EIA but the introduction is confusing in relation to the aim of the paper.

 The methods are not clearly explained. The paper refers to “EIA tools” but it does not describe what tools, and only provides a link to an online questionnaire and online templates for each step of the impact assessment. It seems step one is based on a CEN standard but for the rest of the steps it is not clear how the tools, which are sometimes an online template for a plan or guidelines (which might not be considered tools) have been built.

 The case studies are not described, only the sector where the NM is going to be used is provided. I am not an expert on ethics but I believe the geographical region and social context where the NM is to be applied influences on the ethical impact.

 The method section is a mixture of the explanation of the step, and the testing performed on it. In my view it would be more clear to have a description of the case studies, a description of the tools and then and explanation on how each step was tested, describe the stakeholders and the number of group discussions had for each step.

 The testing of the “EIA tools” is not clear. 

 More detailed comments are below:

**Introduction:**

- There is a bit of repetition on the aims of the paper “This paper reports on test of guidelines…” and later on it repeats “the present paper reports on test and stakeholder feedback….”.  Then the last paragraph indicates “the aim is to give a step-by-step...”.
- Please indicate clearly in one paragraph what is the aim of the paper. How that was achieved can be moved to the Methods section.

**Methods:**

- Malsch et al. (pending publication). Even if the book is not been published yet, please indicate the provisional title and the editorial so when it is available the readers can search for it.
- OECD 2014: please explain briefly what was the aim of the discussion.

**Step 1: screening potential ethical impacts:**

- There is a lack of clarity on what you mean by “tool”. In the first paragraph you refer to the CEN standard as a tool “making the tool suitable for screening” but in the second paragraph you indicate you develop a tool “online tool developed by us” based on the standard.
- It would be better just to refer to the CEN standard as a “standard” and indicate that the project (please delete “by us”) implemented the standard into an online tool.
- Please describe the field of expertise of the “nanosafety experts”; were they ethic experts on emerging technologies, toxicologist, risk assessors? Describe what material or description was given to them to judge so Table 1 makes sense.
- Refer to nanoCOMOMONS as an “EU H2020 project…”.
- Explain who were the three groups of stakeholders (industry, NGOs?).

**Discussion on improvements of the EIA tools:**

- This section does not really discuss the potential improvements based of the testing explained in the previous sections. It adds new information on new discussions.

Is the rationale for developing the new method (or application) clearly explained?

No

Is the description of the method technically sound?

Partly

Are the conclusions about the method and its performance adequately supported by the findings presented in the article?

No

If any results are presented, are all the source data underlying the results available to ensure full reproducibility?

No

Are sufficient details provided to allow replication of the method development and its use by others?

No

Reviewer Expertise:

Exposure and risk assesment of chemicals including nanomaterials, Safe and sustainable by desing

I confirm that I have read this submission and believe that I have an appropriate level of expertise to state that I do not consider it to be of an acceptable scientific standard, for reasons outlined above.

# Response

**Published:** 2024-01-10

**Authors:** Neelina Hermina MALSCH

Thank you for the comments. The aims of the paper and intended users of the EIA methodology were clarified in the introduction, short descriptions of case studies were added and the explanation of the six-step EIA procedure was separated from results of testing it on the case studies.  Info on OECD 2014 was added, and the reference to the book chapter on the water case study was updated, it will be published in March 2024. To clarify the terminology: The CEN CWA is a standard, the RiskGONE deliverable D3.6 are the guidelines and the online decision trees and forms in the cloud are tools: https://www.enaloscloud.novamechanics.com/riskgone/EIA/ More information on the backgrounds of the nanosafety experts participating in discussions of cases and the EIA online tools is included at the appropriate place. Since the responses were given anonymously and the events were attended by people from several disciplines, it is not possible to specify the specialisations. The participants in all events were mostly academics from different parts of the world. The participants in the training organised in the H2020 project NanoCommons responded to a case study of nanomaterials in dentistry. The presentation of the case is still accessible online in this video (see also endnote 3): https://www.youtube.com/watch?v=Ek2oQS_95VY&t=4s What is relevant in the context of the present methodology paper is not the specifics of the case, but the discrepancy between the estimated severities between the participants and the ethicist, and between the three groups. This is a recurrent phenomenon. The title of the section "Potential improvements of the EIA tools" was changed to "Reports of stakeholder discussions of improvements of the EIA tools" to clarify the contents.
